# Supplementary material for: ABCC4 impairs the clearance of plasma LDL cholesterol through suppressing LDLR expression in the liver
Source: Commun Biol. 2025 Oct 2;8:1414. doi: 10.1038/s42003-025-08818-x (PMC12491593; doi:10.1038/s42003-025-08818-x)
Supplement: Supplementary file 2 — Supplementary information [file 42003_2025_8818_MOESM2_ESM.pdf]

Supplementary information

Title: ABCC4 impairs the clearance of plasma LDL cholesterol through suppressing LDLR expression in the liver

Authors: Jiaxin Chen [#1, 2], Hui Huang[# 2], Chi Chen[# 2], Guofang Xia[# 1], Hao Huang[2], Yan Xiong[2], Peng Luo[2], Yu Chen[2], Jinsong Li[2], Liang Wen[2], Lu Li[2], Jing Lin[2], Guangre Xu[4], Chenzhang Ji[3], Wenjie Tian[2], Jin Zhou[5], Peng Wei[1], Chengxing Shen[1], Xiaoqing Wang[1, 2]

Supplementary Figures: Fig S1-S10

Supplementary Tables: Table S1-S4

Figure S1 | CRISPR screen identifies ABCC4 as a negative regulator of hepatic LDL receptor.

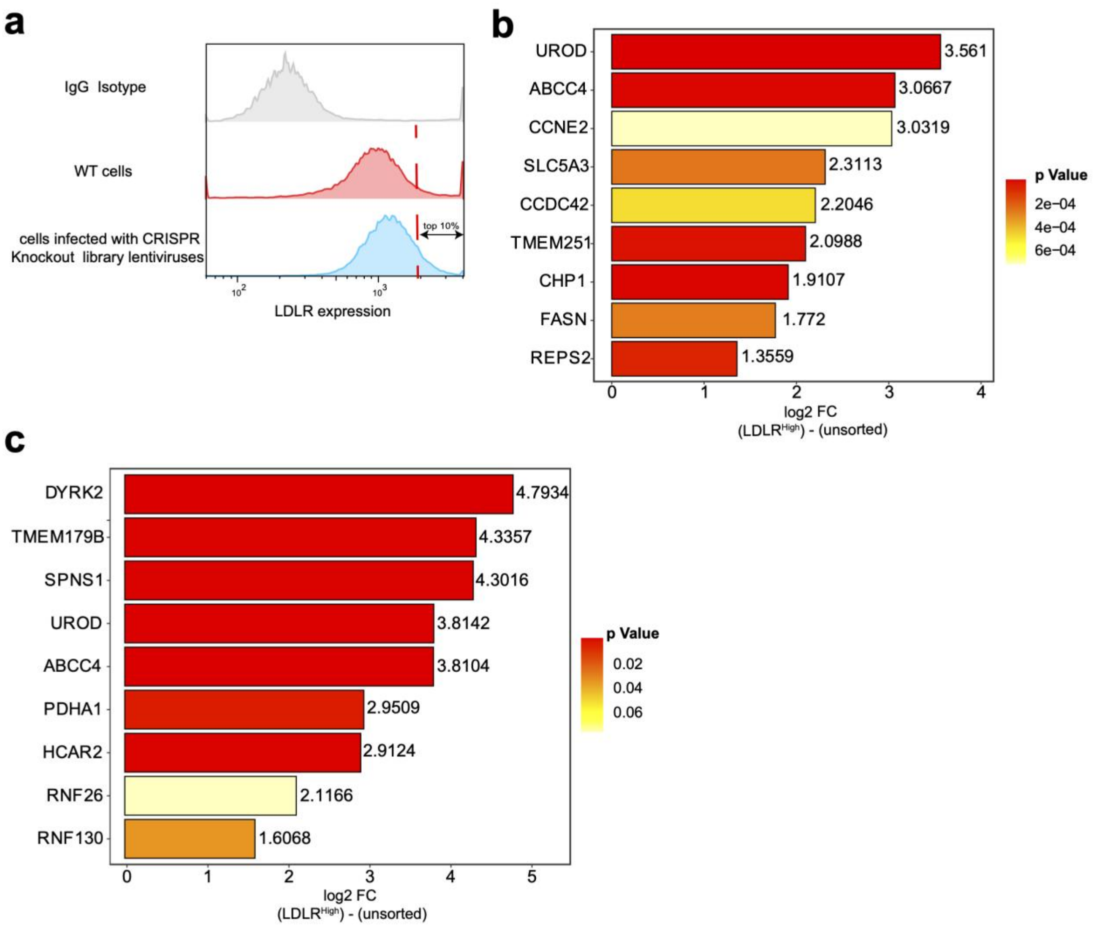

a Plot illustrating the criteria for isolating the LDLR^{high} subpopulation (top 10% with the highest LDLR expression) by FACS. **b** Bar plot displays the $\log_2$ fold-change (FC) of the top-scoring genes in LDLR^{high} subpopulation versus unsorted cells in the screening of M1 part. **c** Bar plot displays the $\log_2$ fold-change (FC) of the top-scoring genes in the LDLR^{high} subpopulation versus unsorted cells in the screening of M2 part.

Figure S2 | ABCC4 depletion increases the cell surface LDLR level independent of its gene expression.

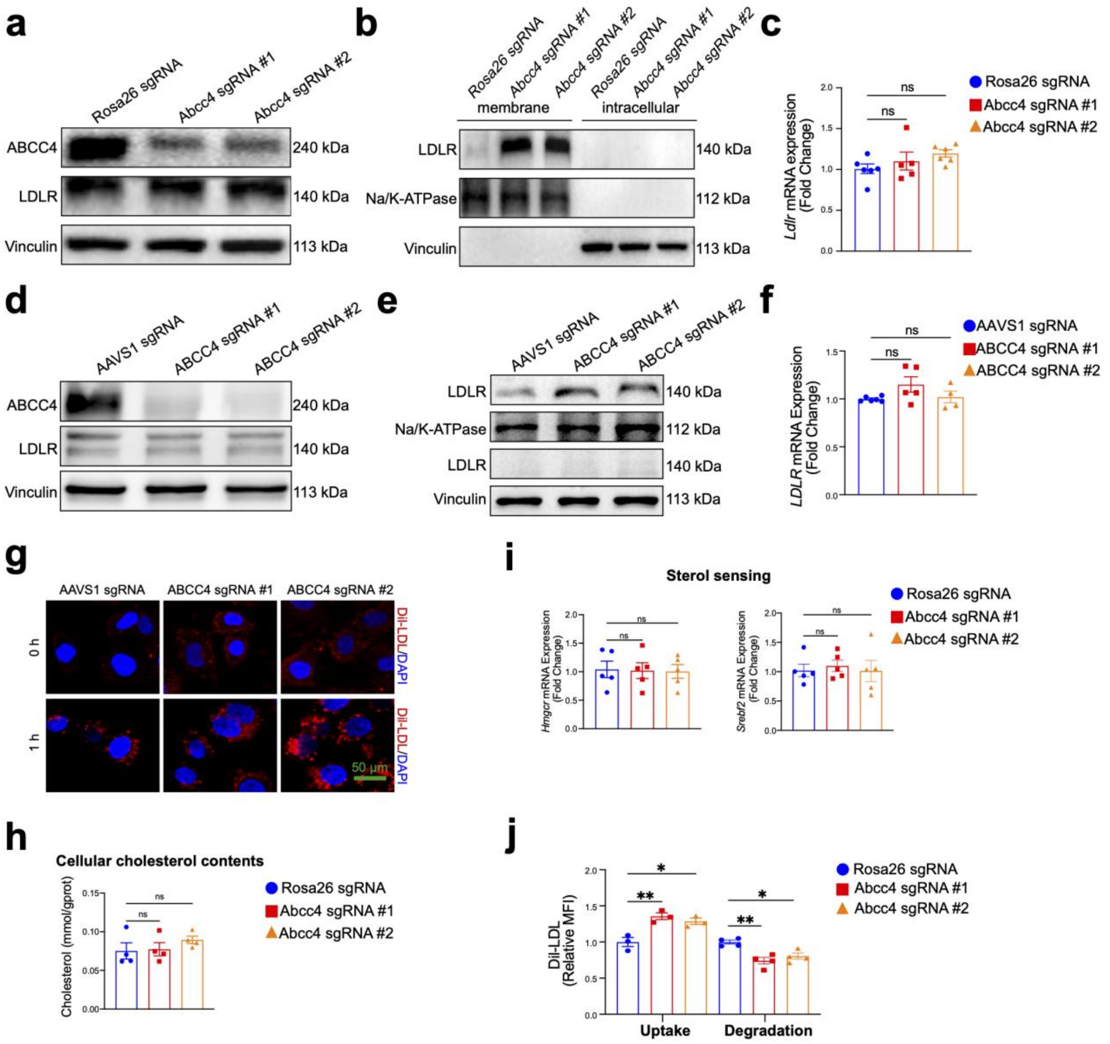

a Immunoblotting analysis of total protein level of LDLR and ABCC4 in AML12 cells (Rosa26 sgRNA, Abcc4 sgRNA #1, Abcc4 sgRNA #2). **b** Immunoblotting analysis of LDLR protein level on the plasma membrane and intracellular fractions in AML12 cells (Rosa26 sgRNA, Abcc4 sgRNA #1, Abcc4 sgRNA #2). **c** RT-qPCR results of *Ldlr* mRNA levels in AML12 cells (Rosa26 sgRNA, Abcc4 sgRNA #1, Abcc4 sgRNA #2) from n = 6 independent experiments. **d** Immunoblotting analysis of total protein level of LDLR and ABCC4 in human hepatocyte cell line LO2 cells (AAVS1 sgRNA, ABCC4 sgRNA #1, ABCC4 sgRNA #2). **e** Immunoblotting analysis of LDLR protein level on the plasma membrane and intracellular fractions in LO2 cells (AAVS1 sgRNA, ABCC4 sgRNA #1, ABCC4 sgRNA #2). **f** RT-qPCR results of *LDLR* mRNA levels in LO2 cells (AAVS1 sgRNA, ABCC4 sgRNA #1, ABCC4 sgRNA #2) from n=6 independent experiments. **g** Representative immunofluorescence images showing that knockout of *ABCC4* in LO2 cells had potentiated influence on DiI-LDL binding (0 h) and uptake (1 h). Blue: DAPI; Red: DiI-LDL. Scale bar: 50 μm. **h** Cellular cholesterol contents in AML12 cells (Rosa26 sgRNA, Abcc4 sgRNA #1, Abcc4 sgRNA #2) from n=4 independent experiments. **i** RT-qPCR results of sterol-sensing genes (*Srebf2*, *Hmgcr*)

in AML12 cells (Rosa26 sgRNA, Abcc4 sgRNA #1, Abcc4 sgRNA #2) from n=5 independent experiments. **j** Flow cytometry analysis of DiI-LDL uptake assay (1 h) and DiI-LDL degradation assay (4 h) in AML12 cells (Rosa26 sgRNA, Abcc4 sgRNA#1, Abcc4 sgRNA#2). The relative MFI of DiI-LDL quantification were from three independent experiments. Statistical analysis was performed by a ordinary one-way ANOVA followed by Bonferroni's multiple comparison test in (**c**), (**f**), (**h**), (**i**), (**j**). $^{*}P \le 0.5$, $^{**}P \le 0.01$, ns: no significance. Data are the mean ± SEM. Source data are provided as a Source Data file.

Figure S3 | C57BL/6 WT male mice were treated with 2×10[11] viral genomes of control AAV_GFP_RNAi (n=6) or AAV_Abcc4_RNAi (n=6) by tail vein injection for 3 weeks.

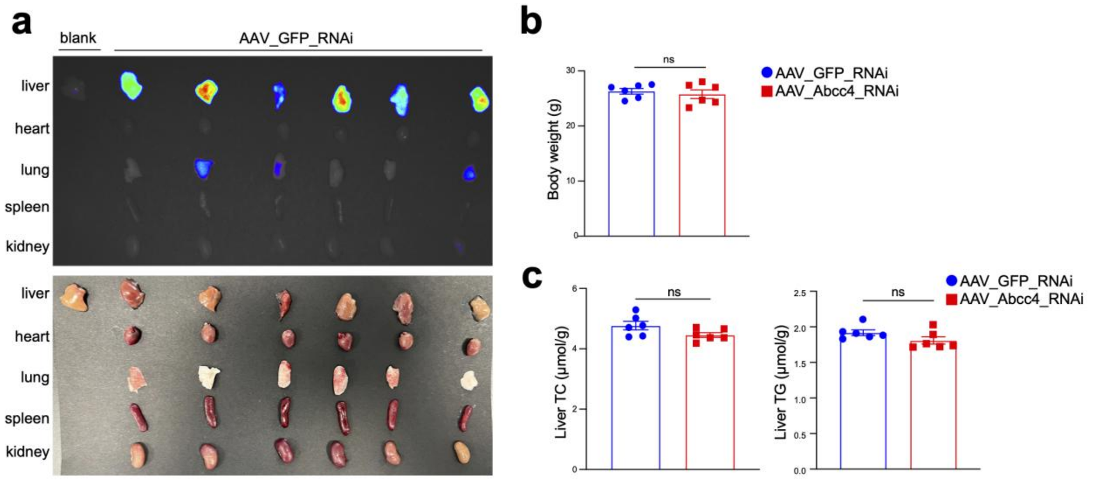

a Male mice (n=6 per group) were injected once at day 0 with either control AAV_GFP_RNAi or AAV_Abcc4_RNAi at a dose of 2×10^{11} viral genomes (VG) per animal. The bioluminescence intensity of green fluorescent protein (GFP) was quantified with a imaging system after 3 weeks. **b** Body weight of mice between two groups. **c** Liver total cholesterol (TC) and triglyceride (TG) levels in WT mice between two groups. Statistical analysis was performed by an unpaired two-tailed Student's t-test in (**b**), (**c**). ns: no significance. Data are the mean ± SEM. Source data are provided as a Source Data file.

Figure S4 | A highly-specific inhibitor of ABCC4, Ceefourin-1 was utilized for the disruption of its function in vitro and in vivo experiments.

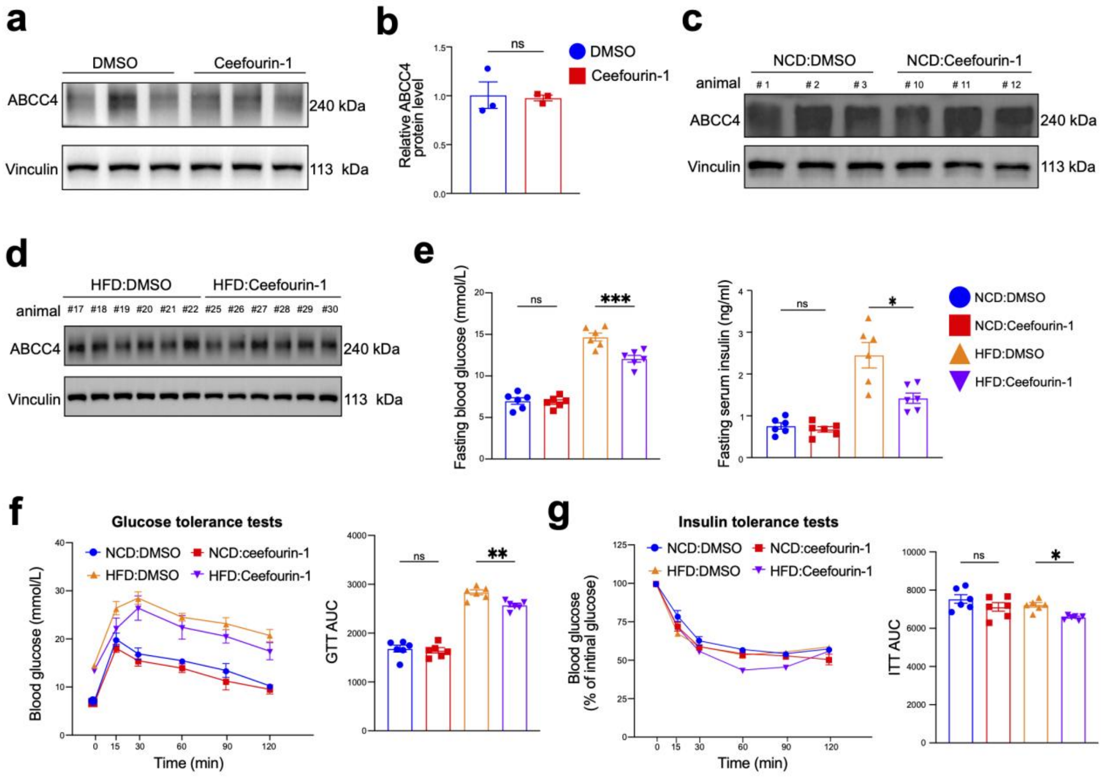

a Immunoblotting analysis of ABCC4 protein expression in AML12 cells treated with DMSO or Ceefourin-1. **b** Quantification of band intensity of ABCC4 protein levels relative to Vinculin (Data from n=3 independent experiments). **c** Immunoblotting analysis of hepatic ABCC4 protein levels in mice administration by Ceefourin-1 (10mg/kg) or vehicle (DMSO and corn oil) for 4 weeks under a NCD condition. **d** Immunoblotting analysis of hepatic ABCC4 protein level in mice administration by Ceefourin-1 (10mg/kg) or vehicle (DMSO and corn oil) for 4 weeks under a HFD condition. **e** Fasting blood glucose and fasting serum insulin levels in mice (n = 6 mice per group). **f** Glucose tolerance tests (GTTs) were measured (n = 6 mice per group). The AUC was used to quantify the GTTs results. **g** Insulin tolerance tests (ITTs) were measured (n = 6 mice per group). The AUC was used to quantify the ITTs results. Statistical analysis was performed by an unpaired two-tailed Student's t-test in (**b**); a ordinary one-way ANOVA followed by Bonferroni's multiple comparison test in (**e**), (**f**), (**g**). *$P \leq 0.5$, **$P \leq 0.01$, *** $P \leq 0.001$, ns: no significance. Data are the mean ± SEM. Source data are provided as a Source Data file.

Figure S5 | The human ABCC4 gene is linked to plasma LDL-C levels and cardiovascular diseases.

a

rsID	*p* value
rs4773884	1.1274e-15
rs7490267	5.1559e-14
rs4771912	2.0996e-7
rs9524917	6.1185e-7
rs10508017	0.000022523
rs34856914	0.000048767
rs4148443	0.000056346
rs2992907	0.000060156

b

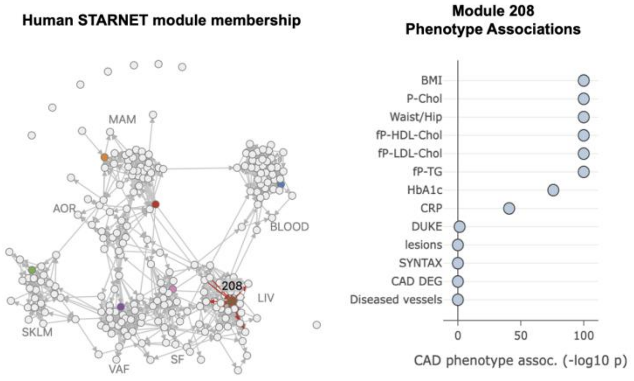

a Table summarizing the variants in the ABCC4 gene locus that are associated with plasma LDL-C levels. Association analyses were conducted using a multivariable-adjusted linear regression model (adjusted for age, sex) in [**a**]. **b** Human gene co-expression network of STARNET data for ABCC4 module membership and cardiometabolic phenotype associations. MAM, free internal mammary artery; AOR, atherosclerotic aortic root; SF, subcutaneous fat; VAF, visceral abdominal fat; SKLM, skeletal muscle; LIV, liver; BMI, body mass index; P-Chol, plasma total cholesterol levels; fP-HDL-Chol, fasting plasma high-density lipoprotein (HDL) cholesterol levels; fP-LDL-Chol, fasting plasma LDL cholesterol levels; fP-TG, fasting plasma triglyceride levels; CRP, C-reactive protein; HbA1c, Hemoglobin A1C; CAD DEG, differentially expressed genes in coronary artery disease; SYNTAX, Synergy between Percutaneous Coronary Intervention with Taxus and Cardiac Surgery;

Figure S6 | Comprehensive transcriptome analysis showed that pathways associated with lipid metabolism were enriched in AML12 cells treated with Ceefourin-1.

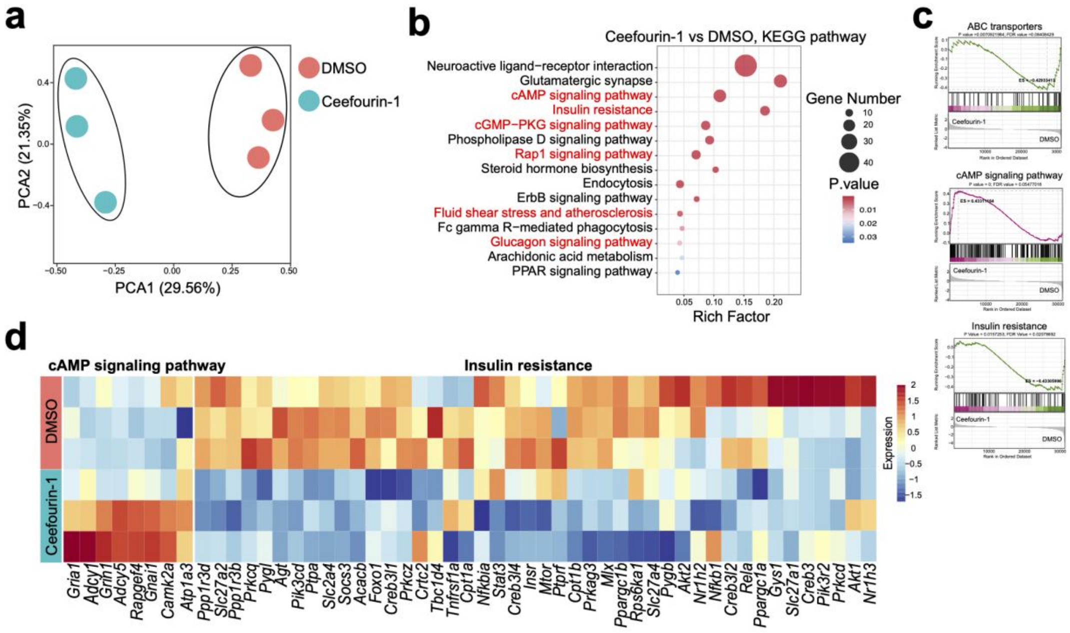

a Principal component analysis (PCA) showing clear separation between AML12 cells treated with vehicle (DMSO) or ABCC4 inhibitor Ceefourin-1. **b** KEGG pathway enrichment analysis of DEGs in AML12 cells between two groups. **c** Gene set enrichment analysis (GSEA) indicating inhibition of ABC transporter pathways, activation of cAMP signaling, and suppression of insulin resistance in Ceefourin-1–treated cells. **d** Heatmap displaying the enriched pathways related to the cAMP signaling and insulin resistance for DEGs in AML12 cells treated with Ceefourin-1.

Figure S7 | Liver-specific disruption and pharmacological inhibition of ABCC4 consistently resulted in decreased PCSK9 protein levels in mice.

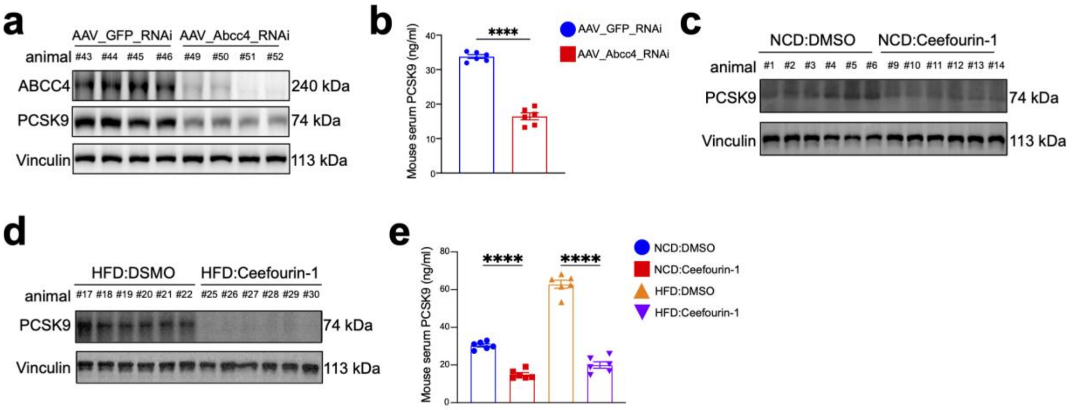

a Immunoblotting analysis of hepatic ABCC4 and PCSK9 protein expressions in WT mice treated with control AAV_GFP_RNAi and AAV_Abcc4_RNAi by tail injection. **b** Serum PCSK9 levels in mice (n=6 per group) treated with control AAV_GFP_RNAi and AAV_Abcc4_RNAi by tail injection. **c** Immunoblotting analysis of hepatic PCSK9 protein levels in mice treated with Ceefourin-1 or vehicle control under a NCD condition. **d** Immunoblotting analysis of hepatic PCSK9 protein levels in mice treated with Ceefourin-1 or vehicle control under a HFD condition. **e** Serum PCSK9 levels in mice (n=6 per group) treated with Ceefourin-1 or vehicle control under a NCD or a HFD condition. Statistical analysis was performed by an unpaired two-tailed Student's t-test in (**b**); a ordinary one-way ANOVA followed by Bonferroni's multiple comparison test in (**e**). **** $P \leq 0.0001$. Data are the mean ± SEM. Source data are provided as a Source Data file.

Figure S8 | Ceefourin-1 and glucagon exerted a synthetic effect on PCSK9 regulation.

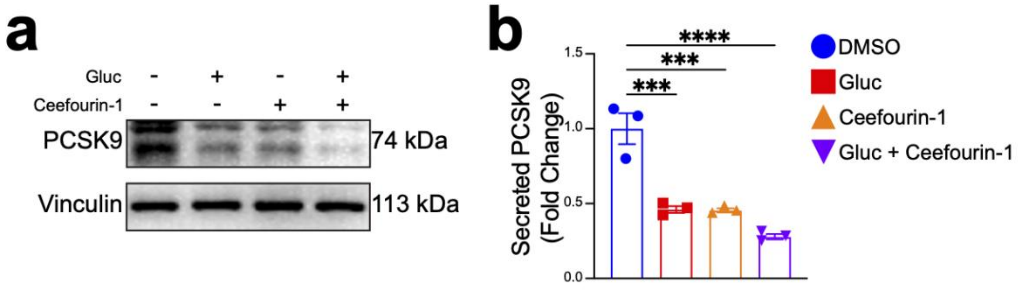

a Immunoblotting analysis of PCSK9 protein levels in AML12 cells treated with Ceefourin-1, glucagon (Gluc) and Ceefourin-1+Gluc. **b** Secreted PCSK9 levels in AML12 treated with Ceefourin-1, glucagon (Gluc) and Ceefourin-1+Gluc (Data from n=3 independent experiments). Statistical analysis was performed by a ordinary one-way ANOVA followed by Bonferroni's multiple comparison test in (**b**). *** $P \le 0.001$, **** $P \le 0.0001$. Data are the mean ± SEM. Source data are provided as a Source Data file.

Figure S9 | Uncropped, unedited immunoblot images of the paper.

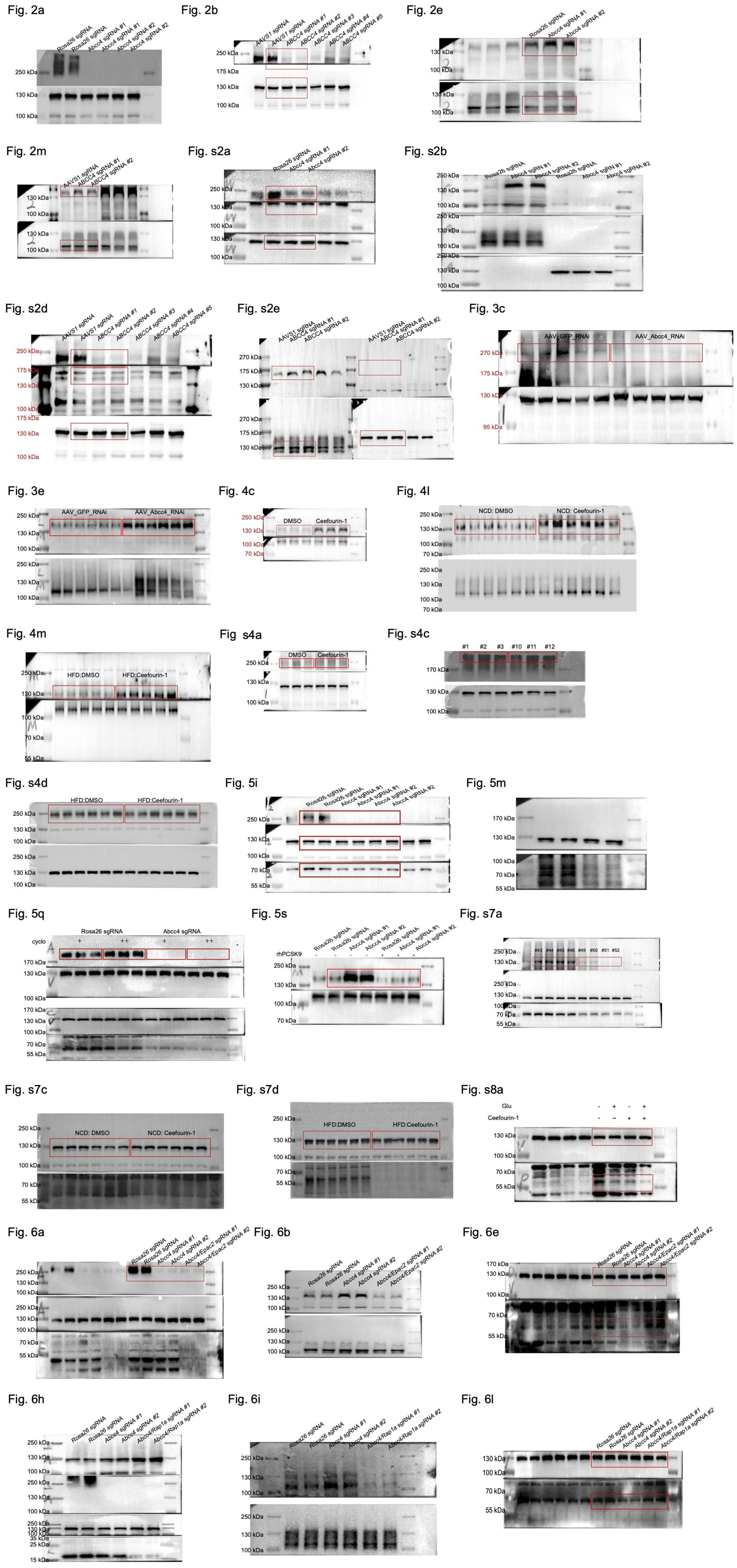

Figure S10 | Gating strategy of flow cytometry (FACS) plots in the paper.

Fig. 2c

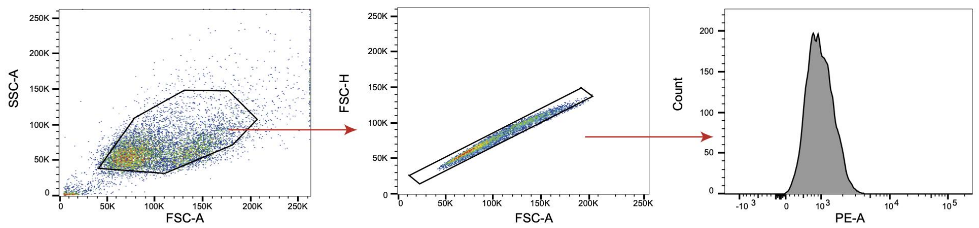

Fig. 2i

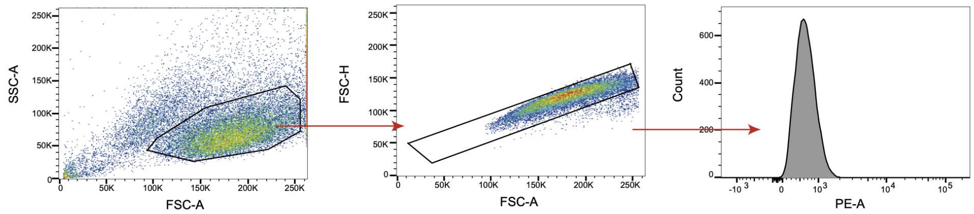

Fig. 2k

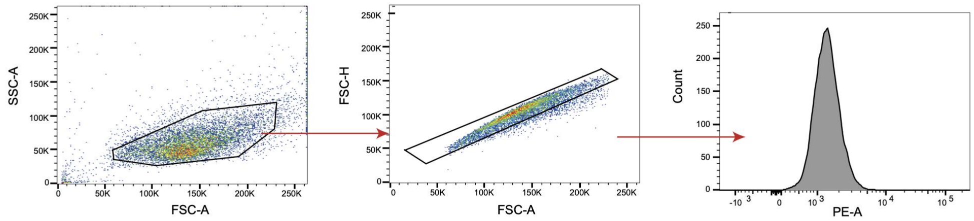

Fig. 2n

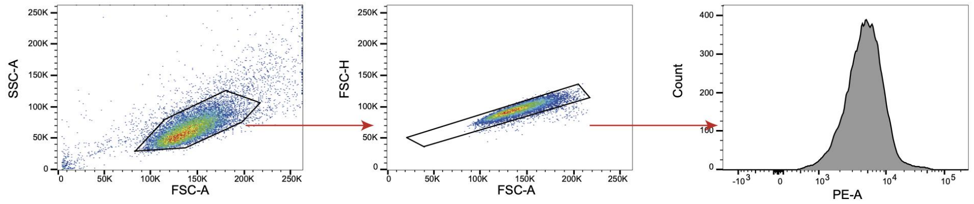

Fig. 4a

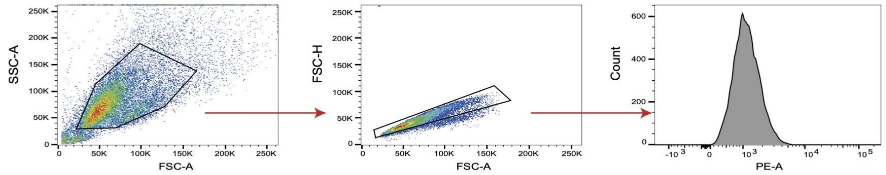

Fig. 6c and 6j

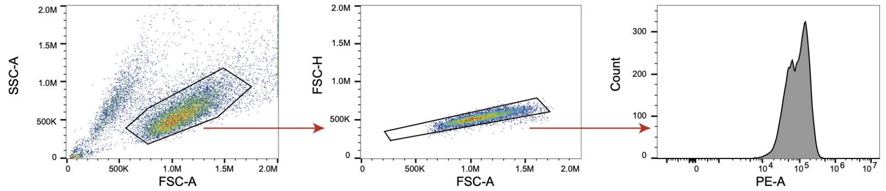

Fig. S1a

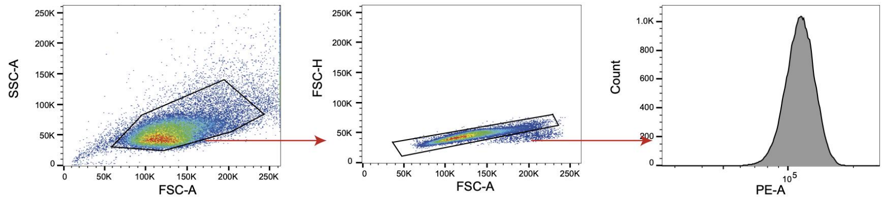

Table S1 | Quality control and Gini index showed an even distribution in the control sgRNA library for the independent screens of M1 part.

	Reads	Mapped	Percentage	Total sgRNAs	Zero counts	Gini Index
Unsorted controls	56163701	42698957	76.03%	92907	3131	0.1331
LDLR^{high} supopulation	19322565	14184939	73.41%	92907	11382	0.2671

Table S2 | Quality control and Gini index showed an even distribution in the control sgRNA library for the independent screens of M2 part.

	Reads	Mapped	Percentage	Total sgRNAs	Zero counts	Gini Index
Unsorted controls	37940348	28381384	74.81%	92907	2687	0.1675
LDLR^{high} subpopulation	14273222	4224051	29.59%	92907	28308	0.5051

Table S3 | Primer sequences

Genes	Forward primer (5'-3')	Reverse primer (5'-3')
Ldlr	TGACTCAGACGAACAAGGCTG	ATCTAGGCAATCTCGGTCTCC
Pcsk9	GAGACCCAGAGGCTACAGATT	AATGTACTCCACATGGGGCAA
Abcc4	AGGAGCTTCAACGGTACTGG	GCCTTTGTTAAGGAGGGCTTC
36b4	GAAACTGCTGCCTCACATCCG	GCTGGCACAGTGACCTCACACG
Hmgcr	AGCTTGCCCGAATTGTATGTG	TCTGTTGTGAACCATGTGACTTC
Srebf2	GCAGCAACGGGACCATTCT	CCCCATGACTAAGTCCTTCAACT
LDLR	TCTGCAACATGGCTAGAGACT	TCCAAGCATTCGTTGGTCCC
GAPDH	GGAGCGAGATCCCTCCAAAAT	GGCTGTTGTCATACTTCTCATGG

Table S4 | single guide RNA sequence for genes

Genes	sequence
Abcc4_sg1	ATGCTGCCGGTGCACACCG
Abcc4_sg2	CCATGGGGAAGACAACCAC
ABCC4_sg1	CCATGGGGAAGACAACCAC
ABCC4_sg2	GGCTGTGATCACACTGCCG
Epac2_sg1	TCTTCACTAGATCGCTCCAA
Epac2_sg2	ATCACCTTCAGATTTGAGG
Rap1a_sg1	AGGAGCAATTTACAGCAATG
Rap1a_sg2	TGTGACTTGGAAGATGAAC
Rosa26_sg	ACTGGAGTTGCAGATCACGA
AAVS1_sg	GTTAATGTGGCTCTGGTTCT
