# Supplementary material for: ABCC4 impairs the clearance of plasma LDL cholesterol through suppressing LDLR expression in the liver
Source: Commun Biol. 2025 Oct 2;8:1414. doi: 10.1038/s42003-025-08818-x (PMC12491593; doi:10.1038/s42003-025-08818-x)
Supplement: Supplementary file 3 — Description of Additional Supplementary files [file 42003_2025_8818_MOESM3_ESM.pdf]

Description of Additional Supplementary files

Supplementary Data 1. The complete list of scores for all 18741 genes were presented in the independent screens of M1 part.

Supplementary Data 2. The complete list of scores for all 18741 genes were presented in the independent screens of M2 part.

Supplementary Data 3. The list of 68 overlapped genes between two independent screenings were identified in the independent screens of M1 part.

Supplementary Data 4. The list of 68 overlapped genes between two independent screenings were identified in the independent screens of M2 part.

Supplementary Data 5. Differentially expressed genes between Rosa26 sgRNA and Abcc4 sgRNA AML12 cells were identified by RNA-seq.

Supplementary Data 6. The source data behind all graphs in the paper.
