# Supplementary material for: ABCC4 impairs the clearance of plasma LDL cholesterol through suppressing LDLR expression in the liver
Source: Commun Biol. 2025 Oct 2;8:1414. doi: 10.1038/s42003-025-08818-x (PMC12491593; doi:10.1038/s42003-025-08818-x)
Supplement: Supplementary file 5 — Reporting summary [file 42003_2025_8818_MOESM5_ESM.pdf]

nature portfolio

Corresponding author(s): Xiaoqing Wang, Chengxing Shen, Peng Wei, Jin Zhou

Last updated by author(s): Aug 20, 2025

Reporting Summary

Nature Portfolio wishes to improve the reproducibility of the work that we publish. This form provides structure for consistency and transparency in reporting. For further information on Nature Portfolio policies, see our Editorial Policies and the Editorial Policy Checklist.

Statistics

For all statistical analyses, confirm that the following items are present in the figure legend, table legend, main text, or Methods section.

n/a	Confirmed	
☐	☒	The exact sample size (n) for each experimental group/condition, given as a discrete number and unit of measurement
☐	☒	A statement on whether measurements were taken from distinct samples or whether the same sample was measured repeatedly
☐	☒	The statistical test(s) used AND whether they are one- or two-sided<br>*Only common tests should be described solely by name; describe more complex techniques in the Methods section.*
☒	☐	A description of all covariates tested
☐	☒	A description of any assumptions or corrections, such as tests of normality and adjustment for multiple comparisons
☐	☒	A full description of the statistical parameters including central tendency (e.g. means) or other basic estimates (e.g. regression coefficient) AND variation (e.g. standard deviation) or associated estimates of uncertainty (e.g. confidence intervals)
☒	☐	For null hypothesis testing, the test statistic (e.g. F, t, r) with confidence intervals, effect sizes, degrees of freedom and P value noted<br>*Give P values as exact values whenever suitable.*
☒	☐	For Bayesian analysis, information on the choice of priors and Markov chain Monte Carlo settings
☒	☐	For hierarchical and complex designs, identification of the appropriate level for tests and full reporting of outcomes
☒	☐	Estimates of effect sizes (e.g. Cohen's d, Pearson's r), indicating how they were calculated

Our web collection on statistics for biologists contains articles on many of the points above.

Software and code

Policy information about availability of computer code

Data collection	Nikon A1 Confocal Microscope (confocal imaging); LightCycler® 96 Instrument (real-time quantitative PCR); BD FACS Melody system (flow cytometry); ChemiDoc Imaging System (immunoblotting); TECAN Microplate readers (protein concentration and ELISA measurements)
Data analysis	Statistical analysis was conducted using Prism 10 (GraphPad Software).

For manuscripts utilizing custom algorithms or software that are central to the research but not yet described in published literature, software must be made available to editors and reviewers. We strongly encourage code deposition in a community repository (e.g. GitHub). See the Nature Portfolio guidelines for submitting code & software for further information.

Data

Policy information about availability of data

All manuscripts must include a data availability statement. This statement should provide the following information, where applicable:

- Accession codes, unique identifiers, or web links for publicly available datasets
- A description of any restrictions on data availability
- For clinical datasets or third party data, please ensure that the statement adheres to our policy

All the data supporting the findings in this study are included in the main article and its supplementary data files. The processed sequencing data in this paper have been deposited in NCBI GEO database (GSE297526). Further information and requests for reagents may be directed to, and will be fulfilled by Prof. Xiaoqing Wang (Xiaoqing_Wang@uestc.edu.cn).

Research involving human participants, their data, or biological material

Policy information about studies with human participants or human data. See also policy information about sex, gender (identity/presentation), and sexual orientation and race, ethnicity and racism.

Reporting on sex and gender	N/A
Reporting on race, ethnicity, or other socially relevant groupings	N/A
Population characteristics	N/A
Recruitment	N/A
Ethics oversight	N/A

Note that full information on the approval of the study protocol must also be provided in the manuscript.

Field-specific reporting

Please select the one below that is the best fit for your research. If you are not sure, read the appropriate sections before making your selection.

☒ Life sciences ☐ Behavioural & social sciences ☐ Ecological, evolutionary & environmental sciences

For a reference copy of the document with all sections, see nature.com/documents/nr-reporting-summary-flat.pdf

Life sciences study design

All studies must disclose on these points even when the disclosure is negative.

Sample size	The sample sizes were chosen based on similar experiments by other groups in the fields. At least six mice were designed for each group in vivo experiments. At least three independent experiments were performed for in vitro assays.
Data exclusions	No samples or animals were excluded from the analyses.
Replication	Numbers of replicates were stated in the figure legends.
Randomization	All samples were randomly allocated to experimental groups.
Blinding	No blinding was performed in this study. Blinding was not feasible because cells were manipulated in various ways according to experimental designs.

Reporting for specific materials, systems and methods

We require information from authors about some types of materials, experimental systems and methods used in many studies. Here, indicate whether each material, system or method listed is relevant to your study. If you are not sure if a list item applies to your research, read the appropriate section before selecting a response.

Materials & experimental systems

n/a	Involved in the study	
☐	☒	Antibodies
☐	☒	Eukaryotic cell lines
☒	☐	Palaeontology and archaeology
☐	☒	Animals and other organisms
☒	☐	Clinical data
☒	☐	Dual use research of concern
☒	☐	Plants

Methods

n/a	Involved in the study	
☒	☐	ChIP-seq
☐	☒	Flow cytometry
☒	☐	MRI-based neuroimaging

Antibodies

Antibodies used	anti-LDLR antibody (1:1000 for immunoblotting, Abmart, T55235);<br>anti-ABCC4 antibody (1:1000 for immunoblotting, Abcam, ab15602);<br>anti-PCSK9 antibody (1:1000 for immunoblotting, Proteintech, 27882-1-AP);

anti-EPAC2 antibody (1:1000 for immunoblotting, Proteintech, 19103-1-AP);
anti-RAP1A antibody (1:1000 for immunoblotting, Proteintech, 16336-1-AP);
anti-Vinculin antibody (1:5000 for immunoblotting, Sigma, V9131);
anti-Na/K-ATPase antibody (1:3000 fro immunoblotting, Abmart, T55159);
anti-mLDLR antibody (1:100 for flow cytometry, R&D Systems, FAB2255P);
anti-human LDLR antibody (1:100 for flow cytometry, R&D Systems, FAB2148P);
HRP Conjugated Goat anti-Rabbit IgG polyclonal Antibody (1:10000 for immunoblotting, huabio, HA1001);
HRP Conjugated Goat anti-Mouse IgG polyclonal Antibody (1:10000 for immunoblotting, huabio, HA1006);
Rabbit Anti-Rat IgG H&L (HRP) (1:10000 for immunoblotting, Abcam, ab6734);

Validation

anti-LDLR antibody (Abmart, T55235)
https://www.ab-mart.com.cn/page.aspx?node=%2077%20&id=%201519

anti-ABCC4 antibody (Abcam, ab15602)
https://www.abcam.cn/products/primary-antibodies/mrp4-antibody-m4i-10-ab15602.html

anti-PCSK9 antibody (Proteintech, 27882-1-AP)
https://www.ptglab.com/products/PCSK9-Antibody-27882-1-AP.htm

anti-EPAC2 antibody (Proteintech, 19103-1-AP
https://www.ptglab.com/products/RAPGEF4-Antibody-19103-1-AP.htm

anti-RAP1A antibody (Proteintech, 16336-1-AP)
https://www.ptglab.com/products/RAP1A-Antibody-16336-1-AP.htm

anti-Vinculin antibody (Sigma, V9131)
https://www.sigmaaldrich.com/US/en/product/sigma/v9131

anti-Na/K-ATPase antibody (Abmart, T55159)
https://www.ab-mart.com.cn/page.aspx?node=%2077%20&id=%201449

anti-mLDLR antibody (R&D Systems, FAB2255P)
https://www.rndsystems.com/cn/products/mouse-ldlr-pe-conjugated-antibody-263123_fab2255p

anti-human LDLR antibody (R&D Systems, FAB2148P)
https://www.rndsystems.com/cn/products/human-ldlr-pe-conjugated-antibody-472413_fab2148p

HRP Conjugated Goat anti-Rabbit IgG polyclonal Antibody (huabio, HA1001)
https://huabio.cn/collections/secondary-antibodies/products/Goat-Anti-Rabbit-IgG-H-L-antibody-HA1001

HRP Conjugated Goat anti-Mouse IgG polyclonal Antibody (huabio, HA1006)
https://huabio.cn/collections/secondary-antibodies/products/Goat-Anti-Mouse-IgG-H-L-antibody-HA1006

Rabbit Anti-Rat IgG H&L (HRP) (Abcam, ab6734)
https://www.abcam.cn/products/secondary-antibodies/rabbit-rat-igg-hl-hrp-ab6734.html

Eukaryotic cell lines

Policy information about cell lines and Sex and Gender in Research

Cell line source(s)

Murine hepatocyte cell line AML12 (Alpha Mouse Liver 12, ATCC CRL-2254) and human embryonic kidney (HEK) 293T cell line (ATCC CRL-3216) were originally obtained from American Tissue Culture Collection Biobank (Manassas, VA, USA). Human hepatocyte cell line LO2 were originally purchased from QuiCell Biotechnology (QuiCell-L090, Shanghai, China).

Authentication

No further authentication of the cell lines was performed before use

Mycoplasma contamination

No test for mycoplasma contamination was performed.

Commonly misidentified lines
(See ICLAC register)

None of these cell lines were utilized.

Animals and other research organisms

Policy information about studies involving animals; ARRIVE guidelines recommended for reporting animal research, and Sex and Gender in Research

Laboratory animals

All animal experimental procedures were approved by the Ethics Committee of Shanghai Sixth People's Hospital Affiliated to Shanghai Jiao Tong University School of Medicine. We have complied with all relevant ethical regulations for animal use. Six-week-old male wide type (WT) C57BL/6 mice were purchased from Shanghai Laboratory Animal Center (SLAC). All mice were euthanized (cervical dislocation) at the end of the experiment, and liver tissues were extracted for further investigation.

Wild animals

This study did not involve wild animals.

Reporting on sex | Male

Field-collected samples | The study did not involve field-collected samples.

Ethics oversight | All animal experimental procedures were approved by the Ethics Committee of Shanghai Sixth People's Hospital Affiliated to Shanghai Jiao Tong University School of Medicine. We have complied with all relevant ethical regulations for animal use.

Note that full information on the approval of the study protocol must also be provided in the manuscript.

Plants

Seed stocks | N/A

Novel plant genotypes | N/A

Authentication | N/A

Flow Cytometry

Plots

Confirm that:

☒ The axis labels state the marker and fluorochrome used (e.g. CD4-FITC).

☒ The axis scales are clearly visible. Include numbers along axes only for bottom left plot of group (a 'group' is an analysis of identical markers).

☒ All plots are contour plots with outliers or pseudocolor plots.

☒ A numerical value for number of cells or percentage (with statistics) is provided.

Methodology

Sample preparation | Cells were washed, trypsinized into single cell suspension, stained with antibody, and analyzed by flow cytometry with appropriate excitation and emission wavelengths following the suggested protocol.

Instrument | BD FACS Melody system

Software | FlowJo™ Software

Cell population abundance | By labeling cells with fluorescently conjugated antibodies against LDLR surface expression, distinct cell subsets can be identified based on their unique antigenic profiles. During flow cytometric analysis, cells are hydrodynamically focused into a single-cell stream and interrogated by lasers, with scattered light and emitted fluorescence signals detected and quantified.

Gating strategy | Gating strategy of the FACS plots are included in Fig. S10.

☒ Tick this box to confirm that a figure exemplifying the gating strategy is provided in the Supplementary Information.
